# Snow occurrence changes over the central and eastern United States under future warming scenarios

**DOI:** 10.1038/srep17073

**Published:** 2015-11-20

**Authors:** Liang Ning, Raymond S. Bradley

**Affiliations:** 1 Key Laboratory of Virtual Geographic Environment of Ministry of Education, School of Geography Science, and Jiangsu Key Laboratory for Numerical Simulation of Large Scale Complex System, School of Mathematical Science, Nanjing Normal University, Nanjing, 210023, China; 2 Northeast Climate Science Center, and Climate System Research Center, Department of Geosciences, University of Massachusetts, Amherst, 01003, United States; 3 Jiangsu Center for Collaborative Innovation in Geographical Information Resource Development and Application, Nanjing, 210023, China

## Abstract

Changes of snow occurrence across the central and eastern United States under future warming for the late 21^st^ century are investigated by applying an empirical hyperbolic tangent function to both observed and downscaled high spatial resolution (~12.5 km) daily temperature and precipitation, to compare the historical (1981–2000) and future (2081–2100) snow occurrence. The observed distributions of snow frequency show that snow-rain transition zones are mainly zonally distributed, since they are largely determined by temperature, with slight shifts to the south over the Appalachian Mountains. The snow-rain transition zone is located around 38–46°N for November and March, and 32–42°N for winter months (DJF). These observed patterns are reproduced well for the historical period by an ensemble average of multiple general circulation models (GCMs). The probabilistic projections show that the snow-rain transition zone will shift to the north under the background of global warming at magnitudes of 2–6 °C, indicating that large areas will experience a partial, or even a very large, loss of snow occurrence in the future. The northward shifts are about 2° latitude under the representative concentration pathways 4.5 (RCP4.5) scenario and 4° latitude under the RCP8.5 scenario. The percentages of the area losing snow occurrence are also assessed.

Increases of global mean surface temperature^1^ have resulted in significant reductions in Northern Hemisphere spring snow cover^2^,^3^,^4^, snowfall amount and high-snow extremes over the U.S.^5^,^6^. in recent decades. Declines in snowfall amount and earlier seasonal melting of snowpack have important consequences for the hydrological cycle and ecosystems, particularly in the regions where water supply is currently dominated by melting snow or ice^7^,^8^,^9^. The frequency of snowfall occurrence, which is mainly determined by surface air temperature^10^, will also probably decrease^11^ given the continued warming projected by the simulations of General Circulation Models (GCMs) for future emissions scenarios^12^. Here we provide an assessment of future changes of the area where precipitation will mainly occur as rain or snow, over the central and eastern U.S. under scenarios of future warming for the late 21^st^ century. These changes will present unique challenges for adapting to and mitigating the effects of climate change on regional water resources, agriculture, transportation, ecosystems, and the economy.

## Results

### Long-term historical verification

**Figure 1:**
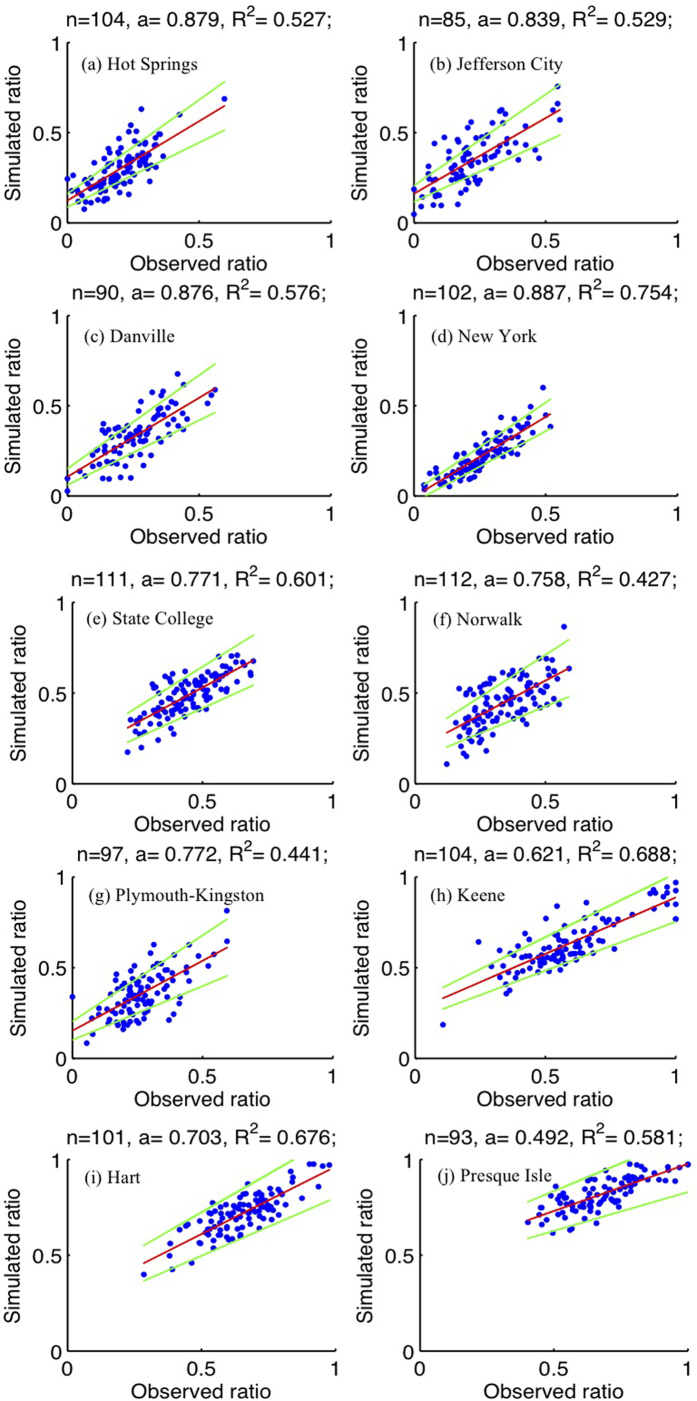
The observed and simulated ratios of snow occurrences over the ten representative stations (blue dots) and the corresponding linear regression (red lines), based on the period 1900–2014. The green lines indicate the 95% confidence intervals. The titles show the sample sizes, slopes of linear regressions, and coefficients of determination.

In a previous study, it was demonstrated that an empirical hyperbolic tangent function can be used to describe the relationship between the snow frequency and temperature^10^ (see Methods section). To verify this method for the central and eastern U.S., we compared observed winter (Nov–Mar) snow occurrences with calculated snow occurrences using observed temperature, for ten representative stations (selected for the historical period, 1900–2014, from the United States Historical Climatology Network (USHCN) data set^13^) (Fig. 1). This data set was used in this part of our analysis because we wanted to verify the relationship over a long period of time. The locations of the ten stations are shown in Fig. S1. These extend across the region affected by seasonal snowfall, and cover different landscapes (e.g. coast, mountain, lakeside, etc.) (see Table S1). The figures are displayed from low-latitude (38°N) to high-latitude (46.65°N) locations. Most slopes of the linear regressions between simulated ratios and observed ratios are close to 1, indicating the empirical hyperbolic tangent function can reasonably reproduce the observed ratios of snow frequency quite well. Usually, the slope magnitude decreases with latitude, with the highest value 0.879 over the station Hot Springs (38°N) and the lowest value 0.492 over the station Presque Isle (46.65°N), indicating slight underestimation.

**Figure 2:**
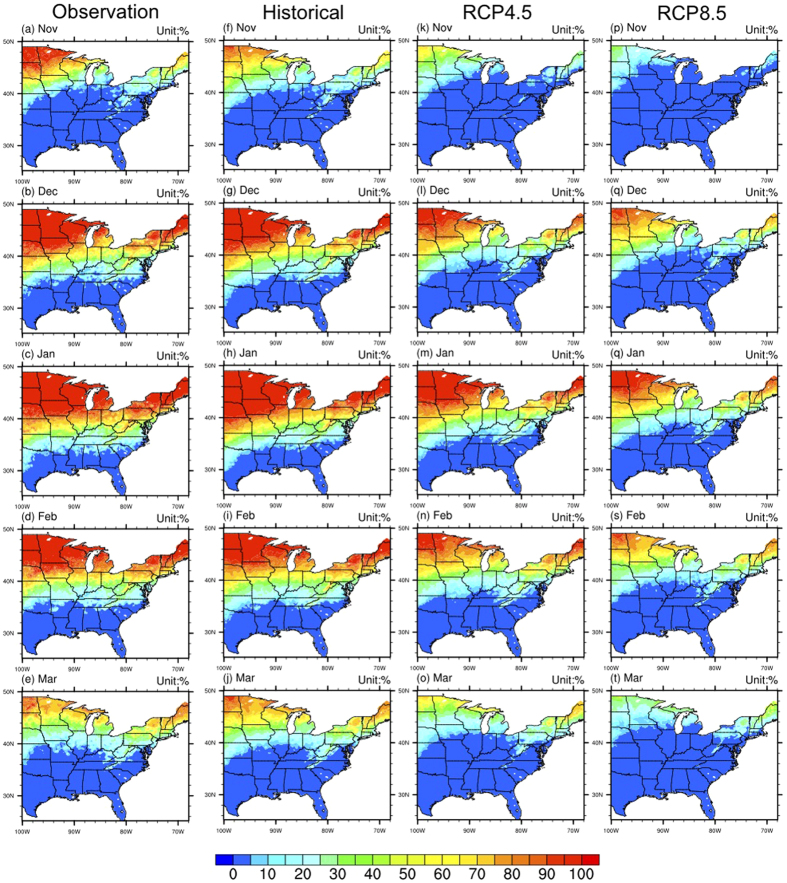
The distributions of ensemble averaged snow frequency from observations (1981-2000) (left column), the simulations under historical (1981–2000) (middle left column), RCP4.5 (2081–2100) (middle right column), and RCP8.5 (2081–2100) (right column) emission scenarios (Unit: %). Maps were generated by NCAR Command Language (NCL).

All equation-calculated snow occurrences can explain more than 40% (*p* < 0.0001) of the total variance of the observed snow occurrences, with most larger than 50%. For the historical time period, over most of the snow/rain transition zone defined as the zone between the rain-dominated area (<10% snow frequency) and snow-dominated area (>90% snow frequency)^11^, the equation-calculated snow frequencies derived by the empirical hyperbolic tangent function can capture both the magnitudes and temporal variations of the observed snow frequencies (Fig. 2). Therefore, the empirical hyperbolic tangent function is a reasonable method to use in estimating the large-scale snow frequency pattern for future climate scenarios.

### Future changes

The observed snow occurrence frequency over the central and eastern U.S. for the period 1981–2000 was estimated by using a high spatial resolution (12.5 km) surface temperature and precipitation data set^14^. The observed spatial patterns of snow frequency for the period 1981–2000 are shown in Fig. 2. For each of the five months with snow (Nov–Mar), there is a transition zone between a snow-dominated region and rain-dominated region. The transition zones are almost zonal, with slight shifts to the south over the western U.S. and Appalachian Mountains, indicating that lower temperatures over the high-elevation region results in larger snow frequencies. For example, during January (Fig. 2c), the transition zone is located around 32–40°N, with a tilt (southerly shift) over the western part and Appalachian Mountains. The location of the transition zones shifts latitudinally during different months, since they are mainly controlled by temperature^10^. January has the coldest temperature, the southernmost location of the transition zone and the largest spatial distribution of snowfall probability; during February (Fig. 2d) and December (Fig. 2b) the transition zone is about 34–42°N, and for March (Fig. 2e) and November (Fig. 2a) it is about 38–46°N. Over the region south of transition zone, there are still some snow occurrences (based on observations), however, since there is very little snow over this region^15^, the biases relative to the application of the empirical function in the current study should be very small.

The simulated snow frequency distributions based on the ensemble averages of 10 GCMs (Table S2) from the Coupled Model Intercomparison Project Phase 5 (CMIP5) archive for the same period (1981–2000) under the historical run^16^ downscaled by Bias-Correction Constructed Analogues (BCCA) method^17^, are similar to observations. In particular, the locations and widths of snow-rain transition zones between the snow-dominated region over the northern part and the rain-dominant region over the southern part are very similar (Fig. 2f–j). The main difference is that the simulated southern tilt during January and February are larger (Fig. 2h,i), and the simulated transition zone during November is wider (Fig. 2f).

By the end of the 21^st^ century (2081–2100) under the representative concentration pathways 4.5 (RCP4.5) and RCP8.5 scenarios^16^, because of warming (Figs S2–S4), the transition zone uniformly shifts to the north during all five months under both scenarios (Fig. 2k–t), indicating that large areas that are currently snow-dominated become rain-dominated in the future. Under representative concentration pathways 4.5 (RCP4.5) scenario, the northern shift of the transition zones is about 2° latitude during December (Fig. 2l), January (Fig. 2m), and February (Fig. 2n), and about 4° latitude during November (Fig. 2k) and March (Fig. 2o). The magnitude of these northern shifts increases to about 5° under the RCP8.5 scenario (Fig. 2p–t). In the future, there will be some areas within the transition zone, or even in the snow-dominated zone of the historical period, that will change to a rain-dominated area, and some snow-dominated areas that change to a mix phase zone, consistent with the observed decreased ratio of snow to precipitation found in previous studies^15^,^18^,^19^. This is because of the northerly shift in the transition zone, especially under RCP8.5, during which most of the central and eastern U.S. becomes rain-dominated. However, it is worth noting that the results shown in Fig. 2 represent climatological averages (20-year means) for future scenarios and so do not exclude the possibility of occasional snow occurrence under scenarios of future warming in the rain-dominated region.

Moreover, since the increases of the daily minimum temperature (Fig. S4) are usually larger than the increases of the daily average temperature (Fig. S2) and daily maximum temperature (Fig. S3), the changes of maximum possible snow occurrences (Fig. S6) are also larger than the changes of minimum possible snow occurrences (Fig. S5) and averaged snow occurrences (Fig. 2). This indicates that the future changes of probability distributions of the snow occurrences are not simply uniform shifts to lower values, but also involve skewness changes towards the lower values.

**Figure 3:**
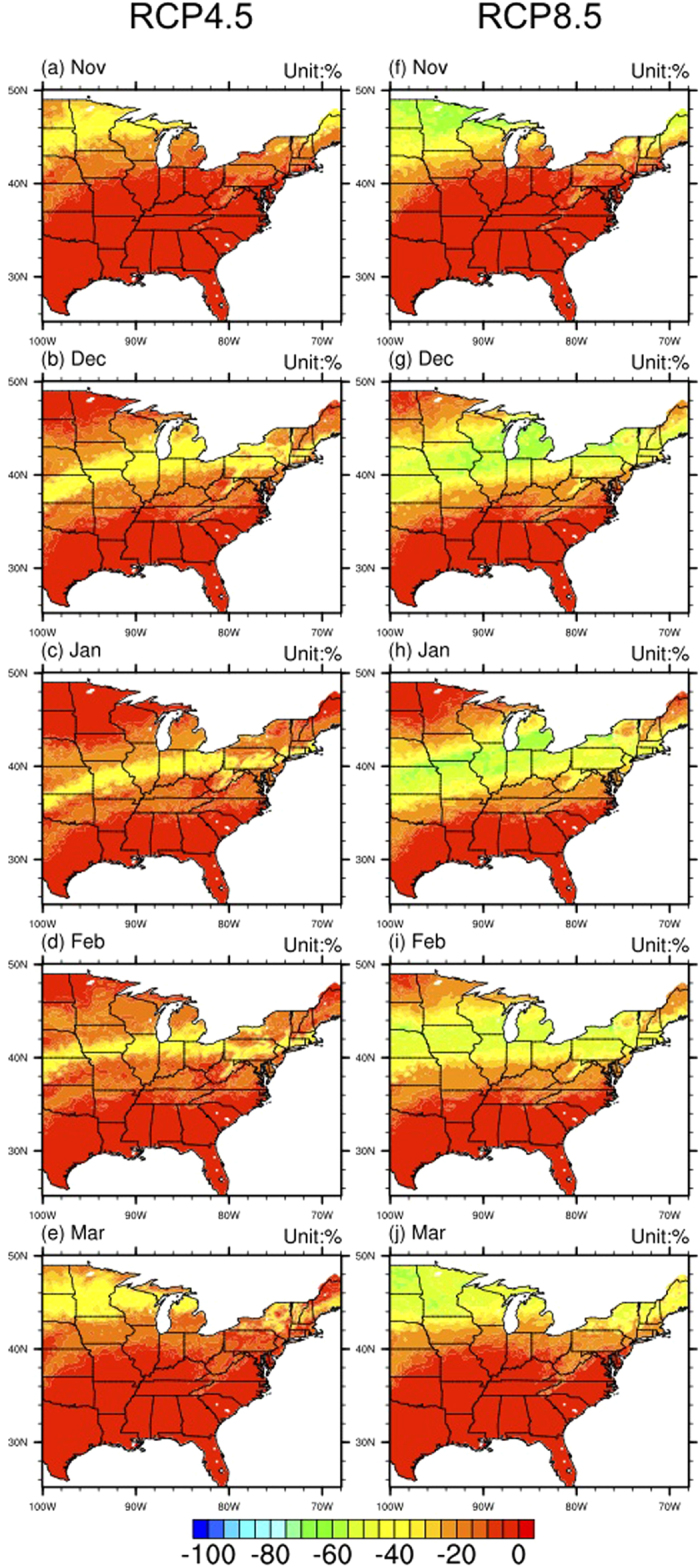
The changes of ensemble averaged snow frequency (RCP scenarios relative to historical simulation) for the RCP4.5 (left column), and RCP8.5 (right column) emission scenarios for the five months (Unit: %). Maps were generated by NCAR Command Language (NCL).

The region with snow frequency reductions is also a zonally-distributed, with a tilt to the south over the western part of the region and in the Appalachian Mountains (similar to the transition zone) usually starting from the southern border of the historical transition zone and extending 10° latitude to the north for December (Fig. 3b), January (Fig. 3c), February (Fig. 3d), under the RCP4.5 scenario, and 12° for November (Fig. 3a) and March (Fig. 3e). The magnitudes of snow frequency reductions range from zero in the north (snow-dominated regions) and the south (rain-dominated regions) to about 80% in the middle, indicating that those previously snow-dominated regions will change to rain-dominated regions. The situations under the RCP8.5 scenario (Fig. 3f–j) are similar, with wider regions of snow frequency reductions (extending 3–4° further north than in the RCP4.5 scenario). The areas of regions with large snow frequency reductions (>80%) are also larger than in the RCP4.5 scenario, indicating more previous snow-dominated regions will change to rain-dominated regions during the higher emission scenario. The magnitudes of snow frequency reductions are also larger under the RCP8.5 scenario over the same area (the region surrounding Chicago shown in Fig. S7 as an example).

**Figure 4:**
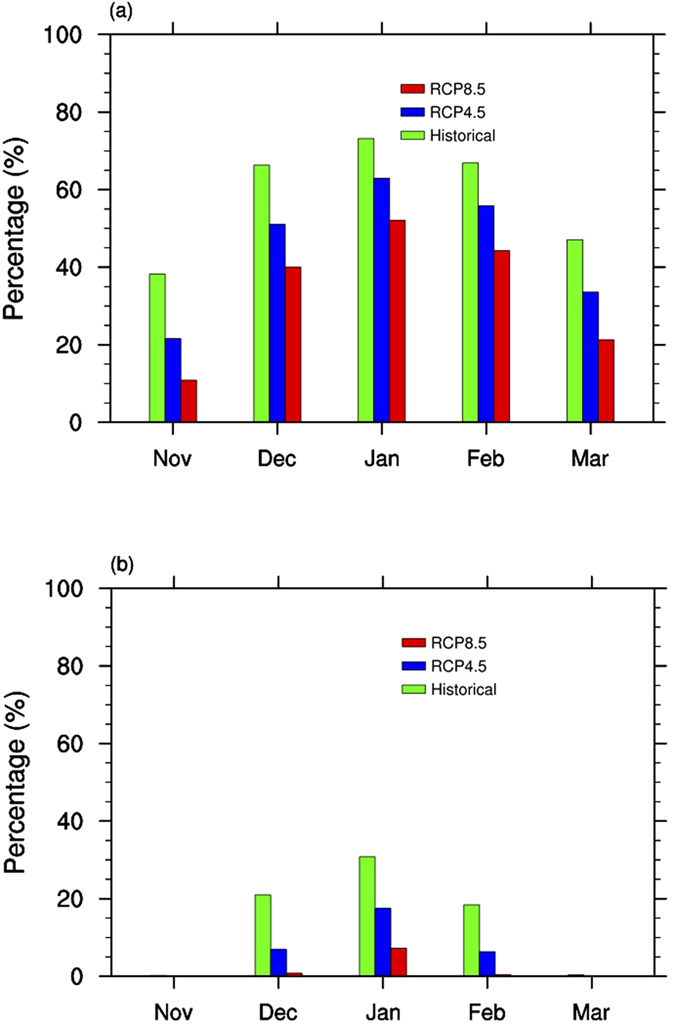
The percentages of area with snow frequency larger than 10% (**a**) and 90% (**b**) under the historical (green), RCP4.5 (blue), and RCP8.5 (red) emission scenarios for the five months (Unit: %).

To quantitatively evaluate the reductions of area with snow occurrence, the percentages of area with snow frequency >10% and >90% under three scenarios are compared in Fig. 4. These two thresholds are chosen because a snow frequency <10% is considered as rain-dominated and a snow frequency >90% is considered as snow-dominated, after taking the biases of empirical equations shown in Fig. 1 into consideration. Under the historical scenario, the region with obvious snow frequency (>10%) accounts for about 37.5%, 67.5%, 72.5%, 67.5%, and 47.5% of the whole central and eastern U.S. for the five months Nov-Mar. These numbers drop to about 22.5%, 50%, 65%, 55%, and 35% under the RCP4.5 scenario, and about 10%, 40%, 52.5%, 45%, and 22.5% under the RCP8.5 scenario (Fig. 4a). On the other hand, under the historical scenario, the region with the largest snow frequency (>90%) accounts for about 1%, 22.5%, 30%, 20%, and 1% of the whole central and eastern U.S. for the five months, and these numbers drop to about 0%, 7.5%, 17.5%, 7.5%, and 0% under the RCP4.5 scenario, and about 0%, 2%, 7.5%, 2%, and 0% under the RCP8.5 scenario (Fig. 4b).

The uncertainties in the changes of area with snow occurrence due to the scenarios, defined as the differences between the two RCP scenarios, are about 10% of the total study region for the main snow season (Fig. 4). These are consistent with the uncertainties in the northern shifts of the transition zone, which have a magnitude about 3° latitude (Fig. 2). For the changes of area with obvious snow frequency (>10%), the inter-GCM uncertainties defined as the standard deviations of changes from all GCMs, are less than 3% of total study region for Nov-Jan and 5–6% for Feb-Mar under the RCP4.5 scenario (Fig. S8a). For the RCP8.5 scenario, the inter-GCM uncertainties are 3% for November, and 3–10% for Dec-Mar. For the changes of area with large snow frequency (>90%), the inter-GCM uncertainties are ~2–5% for mid-winter months in both RCP scenarios (Fig. S8b).

## Conclusions

Future changes of snow occurrences over the central and eastern U.S. under two emission scenarios were investigated using high spatial resolution downscaled ensemble averages from ten GCMs. By the end of this century, due to the increases of surface air temperature, the snow-rain transition zone will shift north by 2–5° latitude, indicating that large areas will have a significant reduction in snowfall occurrence (~15% of the total domain for RCP4.5 scenario and ~25% for RCP8.5 scenario). During November and March, non-negligible snow occurrence (>10%), disappears across most of the region, except for the extreme northern and northwestern areas. This means the snow season over most of the region will become shorter, from five to about three months, consistent with observed shortening of the snow season duration over recent decades^20^. Therefore, the observed reductions of snow cover^2^,^3^ and snowfall amount^5^,^6^ will continue and even amplify through this century. This shortening of the winter snow season and early melting of the snowpack will have serious impacts on water resources, ecosystems, and the economy of this region, especially over the Great Lakes Region^21^. For the region within the transition-zones, e.g., the center of the study region, these results can be used to estimate future influences of snow occurrences (especially night-time snowfall events) with implications for municipal administration (e.g., school closure and traffic interruptions), and the corresponding future planning strategies. These results will also help improve the predictability of future changes of regional water resources, ecosystems, agriculture, and winter recreation activities over the central and eastern U.S., and also help resource-managers and decision-makers prepare corresponding adaptation plans for future warming.

## Methods

The observed and simulated snow frequencies were calculated using the empirical tangent function^10^:


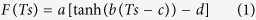


which is based on a larger sample size of global weather stations, updated with longer observations compared with earlier studies^22^,^23^. In equation (1), a, b, c, and d are parameters estimated, based on a larger sample size of global weather stations, and in this study, a = −48.2292, b = 0.7205, c = 1.1662, d = 1.0223^10^.

To verify the empirical hyperbolic tangent function using the long-term USHCN data, the monthly maximum temperature, minimum temperature, and average temperature on days with precipitation, for each of the five months (Nov-Mar) was first calculated. Then, the corresponding snow frequencies were calculated using the empirical hyperbolic tangent function. In this step, to limit the influence of missing values, only those years with at least three months that include more than five days with precipitation were used.

Snow frequency for the period 1981–2000 was estimated by using a high spatial resolution (12.5 km) surface temperature and precipitation data set^14^, (this data set was recently improved to a new version, with a higher spatial resolution (1/16°) and longer time period^24^). The simulated historical and future snow occurrence frequencies were calculated using downscaled temperature and precipitation data with the same resolution based on 10 GCMs (Table S2) from the Coupled Model Intercomparison Project Phase 5 (CMIP5) archive for the period 1981–2000 under the historical scenario, and 2081–2100 under the representative concentration pathways 4.5 (RCP4.5) and RCP8.5 scenarios^15^. The daily maximum temperature and minimum temperature were used to generate the lower limits and upper limits of the snow occurrence distributions, separately. Then, average values of snow occurrences were calculated as weighted averages of snow occurrences related to daily maximum temperature, minimum temperature and average temperature. In this step, since there are only a few missing days, the threshold of picking a month was changed to two days with precipitation.

## Additional Information

**How to cite this article**: Ning, L. and Bradley, R. S. Snow occurrence changes over the central and eastern United States under future warming scenarios. *Sci. Rep.* **5**, 17073; doi: 10.1038/srep17073 (2015).

## Supplementary Material

Supplementary Information
