# Supplementary material for: Snow occurrence changes over the central and eastern United States under future warming scenarios
Source: Sci Rep. 2015 Nov 20;5:17073. doi: 10.1038/srep17073 (PMC4653631; doi:10.1038/srep17073)
Supplement: Supplementary Information [file srep17073-s1.pdf]

Snow occurrence changes over the central and eastern United States under future warming scenarios

Liang Ning[1,2,3*] and Raymond S. Bradley[2]

[1]Key Laboratory of Virtual Geographic Environment of Ministry of Education, School of Geography Science, and Jiangsu Key Laboratory for Numerical Simulation of Large Scale Complex System, School of Mathematical Science, Nanjing Normal University, Nanjing, 210023, China

[2]Northeast Climate Science Center, and Climate System Research Center, Department of Geosciences, University of Massachusetts, Amherst, 01003, United States

[3]Jiangsu Center for Collaborative Innovation in Geographical Information Resource Development and Application, Nanjing, 210023, China

[*] lning@geo.umass.edu

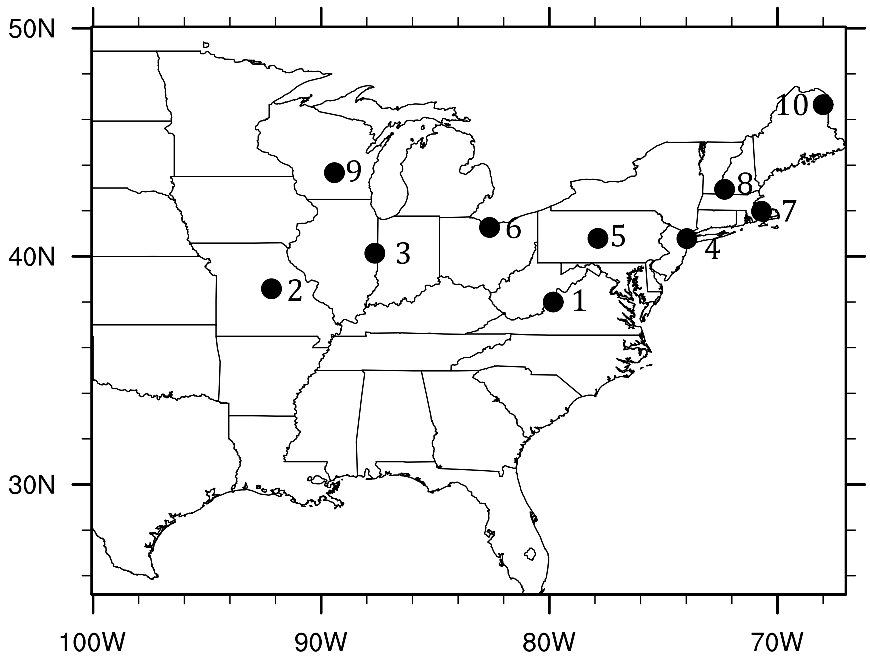

Fig. S1 The locations of the ten representative stations used in this study

Map was generated by NCAR Command Language (NCL).

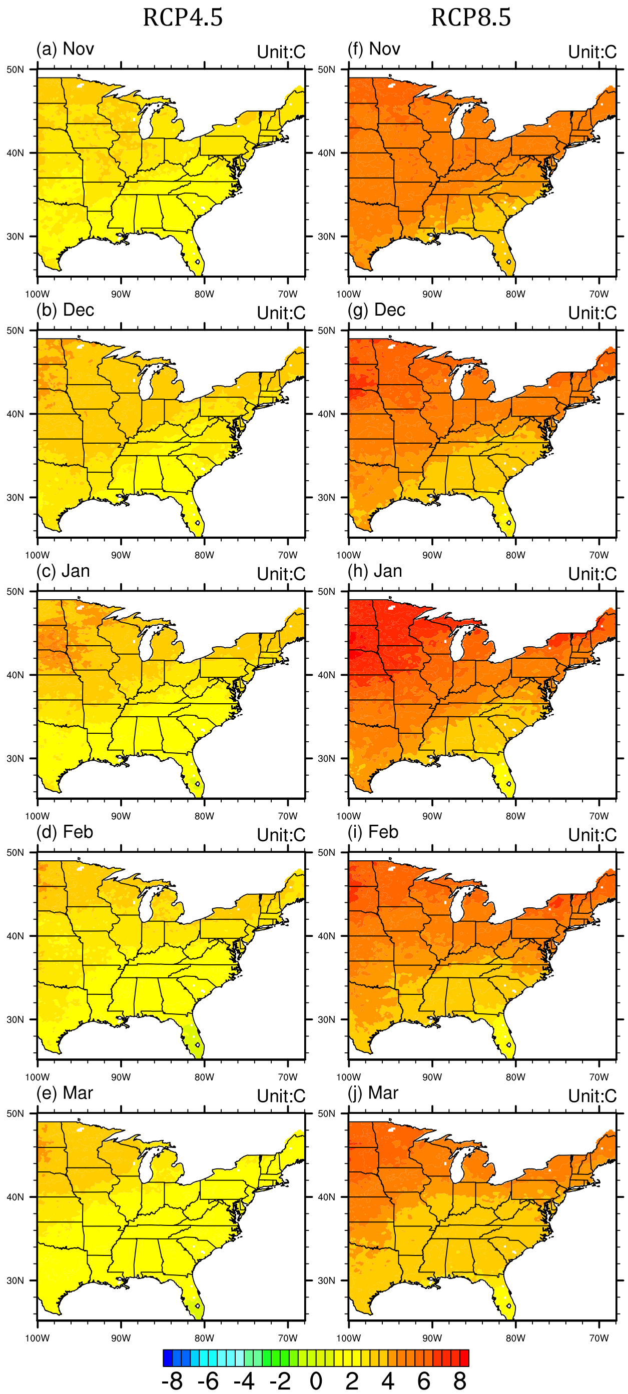

Fig. S2 The changes of ensemble averaged daily average temperature for the RCP4.5 (left column), and RCP8.5 (right column) scenarios for the five months (Unit: °C) Maps were generated by NCAR Command Language (NCL).

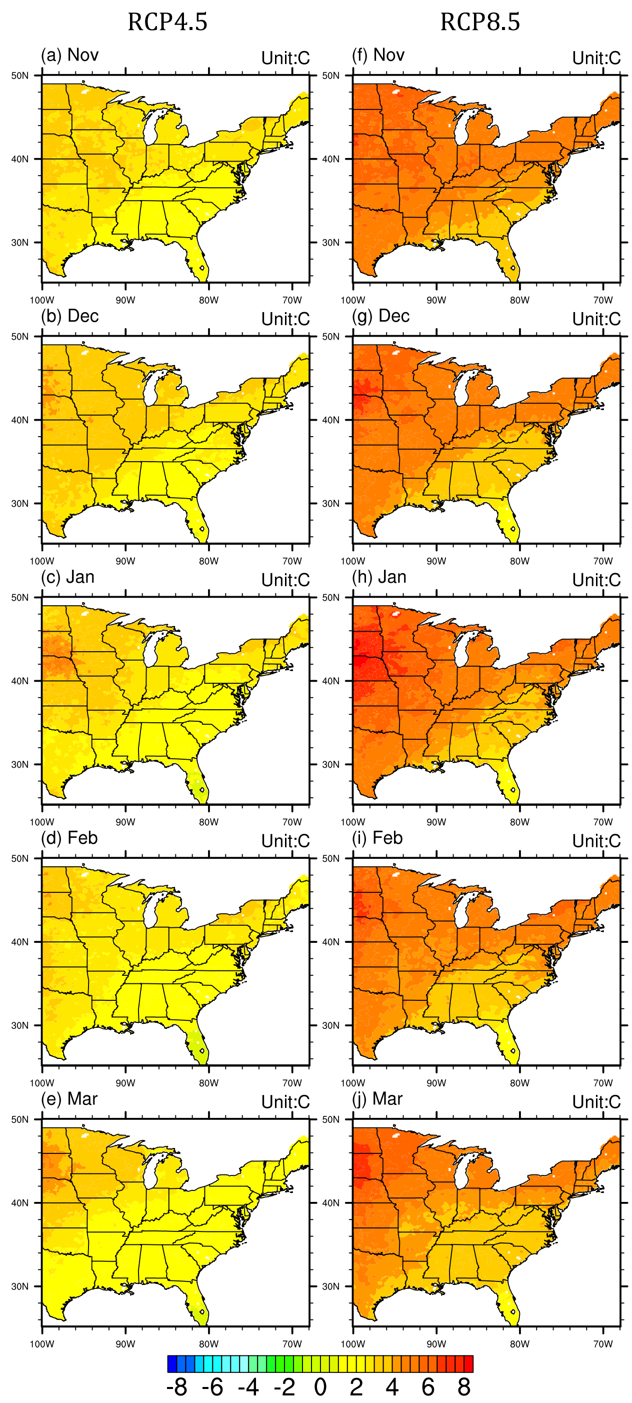

Fig. S3 The changes of ensemble averaged daily maximum temperature for the RCP4.5 (left column) and RCP8.5 (right column) scenarios for the five months (Unit: °C) Maps were generated by NCAR Command Language (NCL).

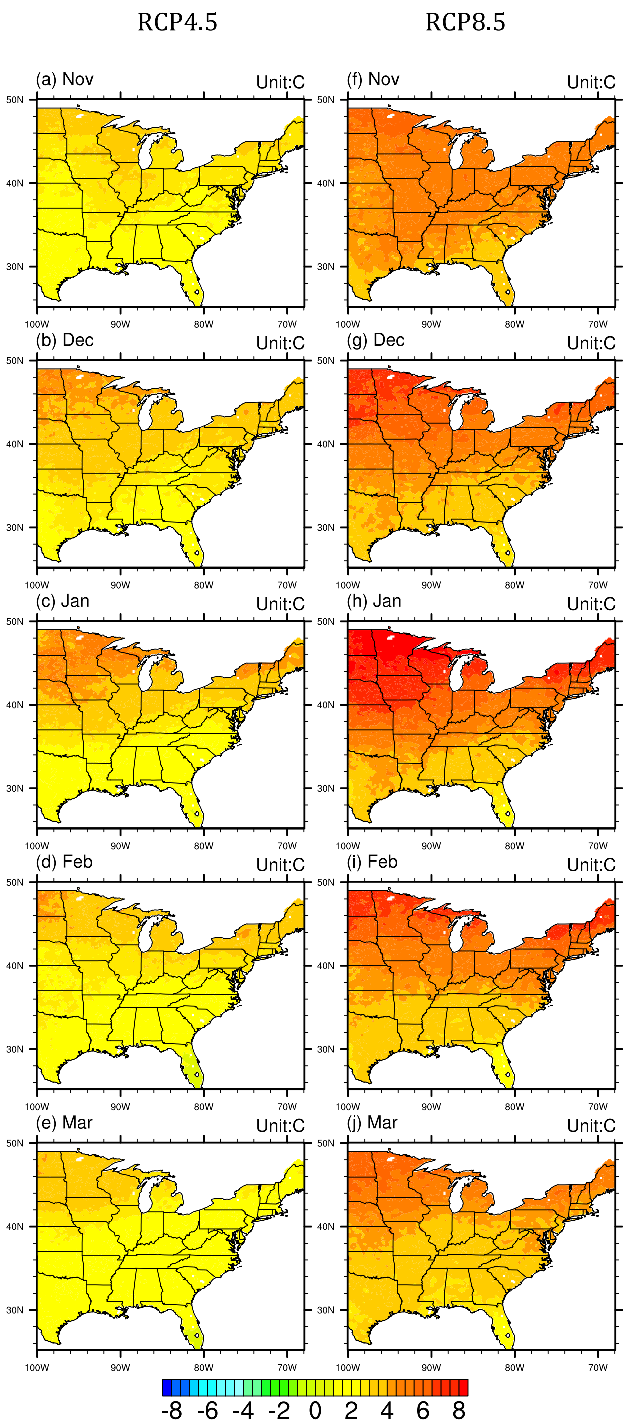

Fig. S4 The changes of ensemble averaged daily minimum temperature for the RCP4.5 (left column) and RCP8.5 (right column) scenarios for the five months (Unit: °C) Maps were generated by NCAR Command Language (NCL).

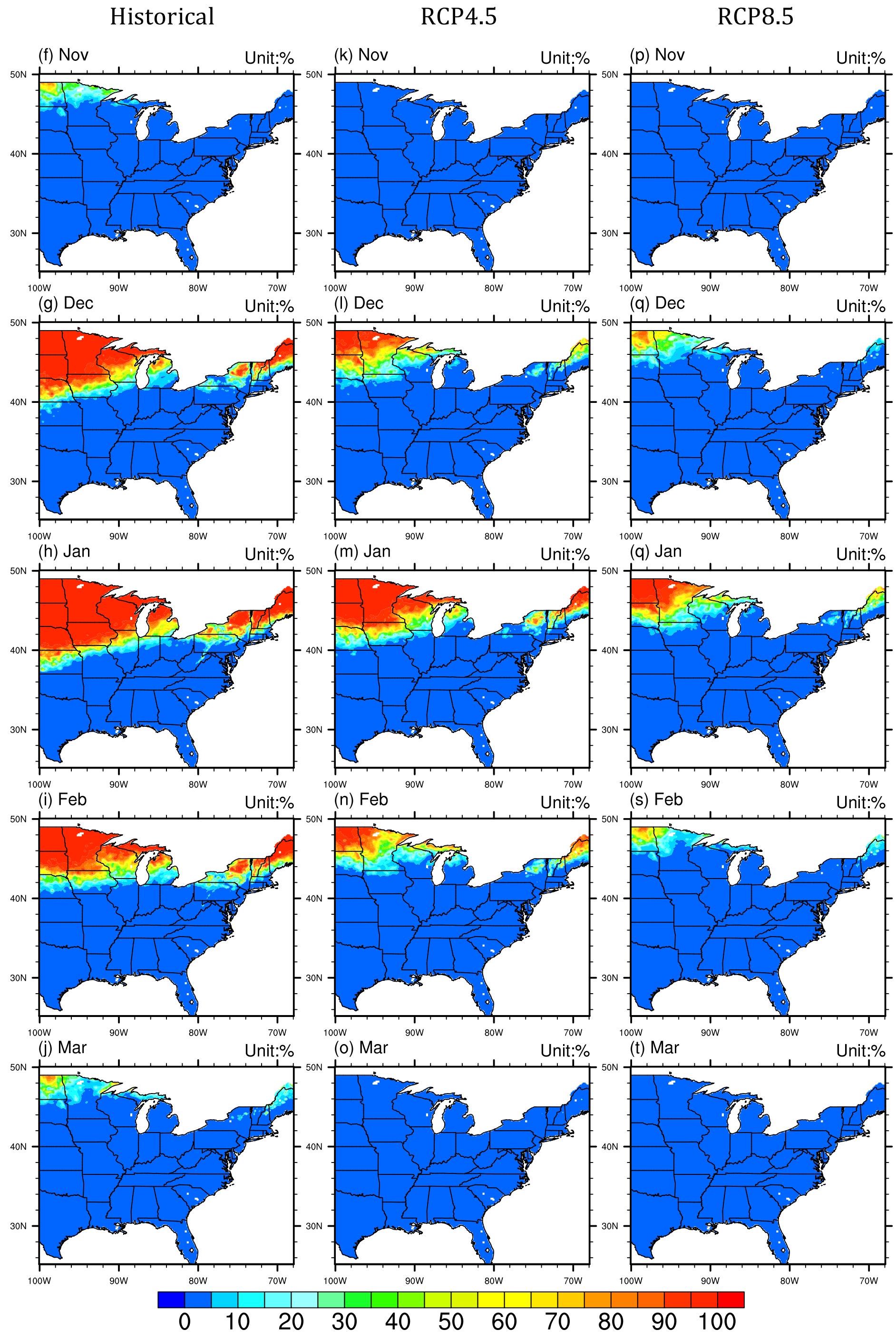

Fig. S5 The distributions of ensemble averaged simulated lower limits of snow occurrences under historical (1981-2000) (left column), RCP4.5 (2081-2100) (middle column), and RCP8.5 (2081-2100) (right column) emission scenarios (Unit: %) Maps were generated by NCAR Command Language (NCL).

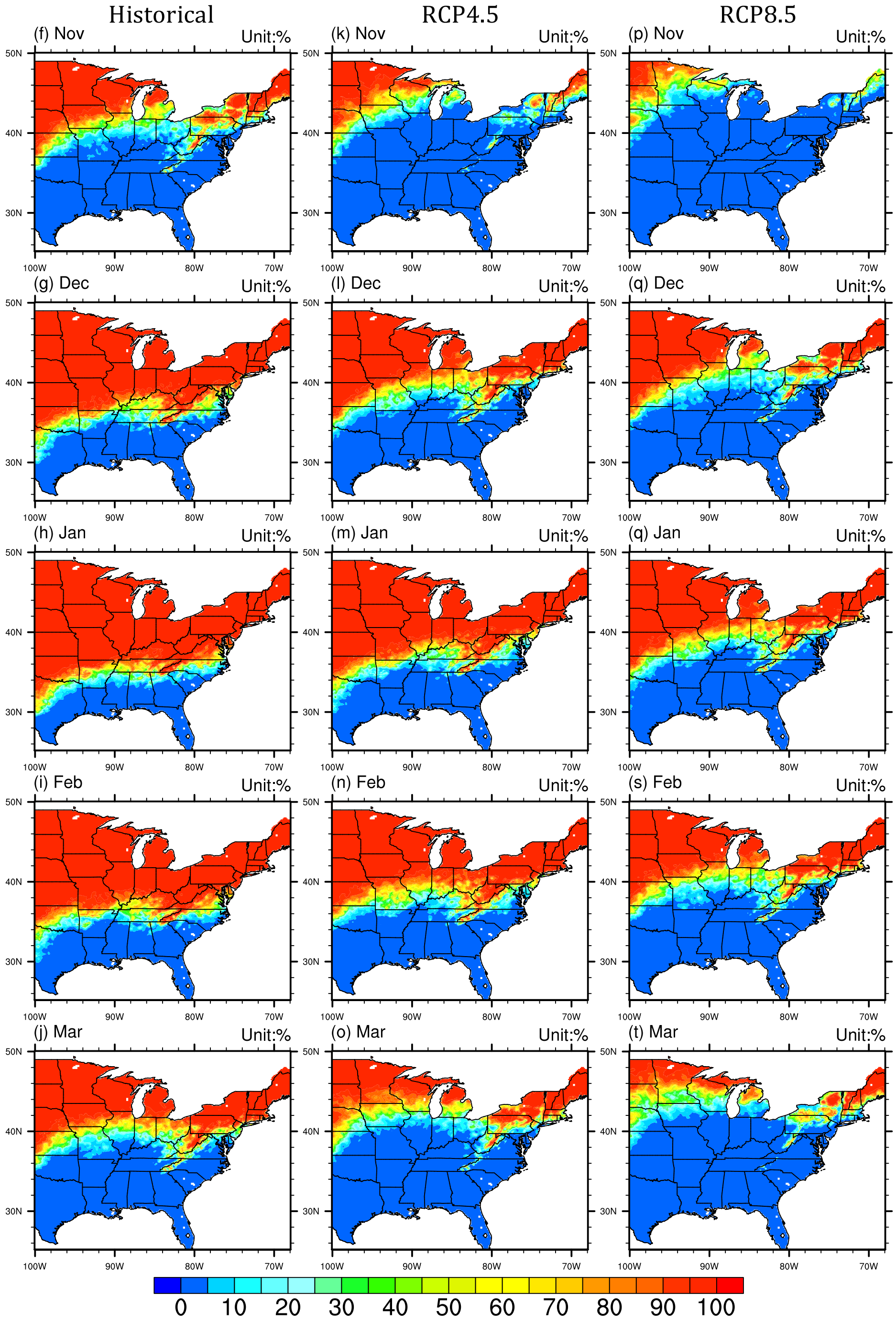

Fig. S6 The distributions of ensemble averaged simulated upper limits of snow occurrence under historical (1981-2000) (left column), RCP4.5 (2081-2100) (middle column), and RCP8.5 (2081-2100) (right column) emission scenarios (Unit: %) Maps were generated by NCAR Command Language (NCL).

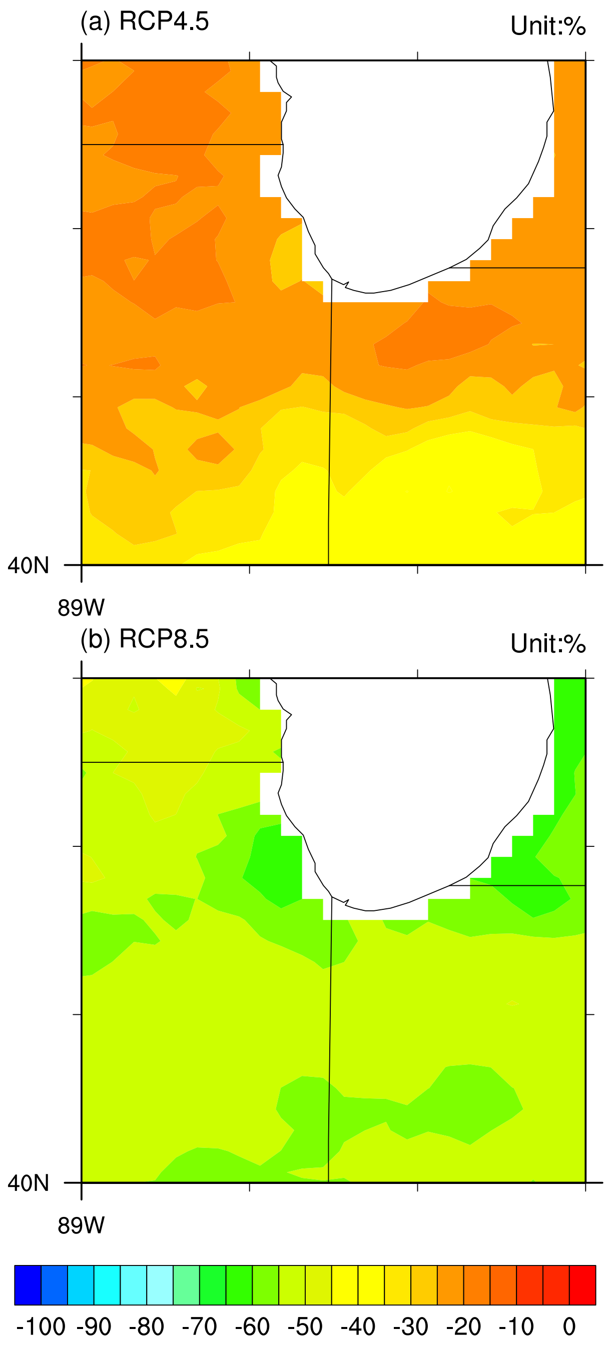

Fig. S7 The changes of ensemble averaged January snow frequency for RCP4.5 (a) and RCP8.5 (b) emission scenarios over the region surrounding Chicago (RCP scenarios relative to historical simulation)

Maps were generated by NCAR Command Language (NCL).

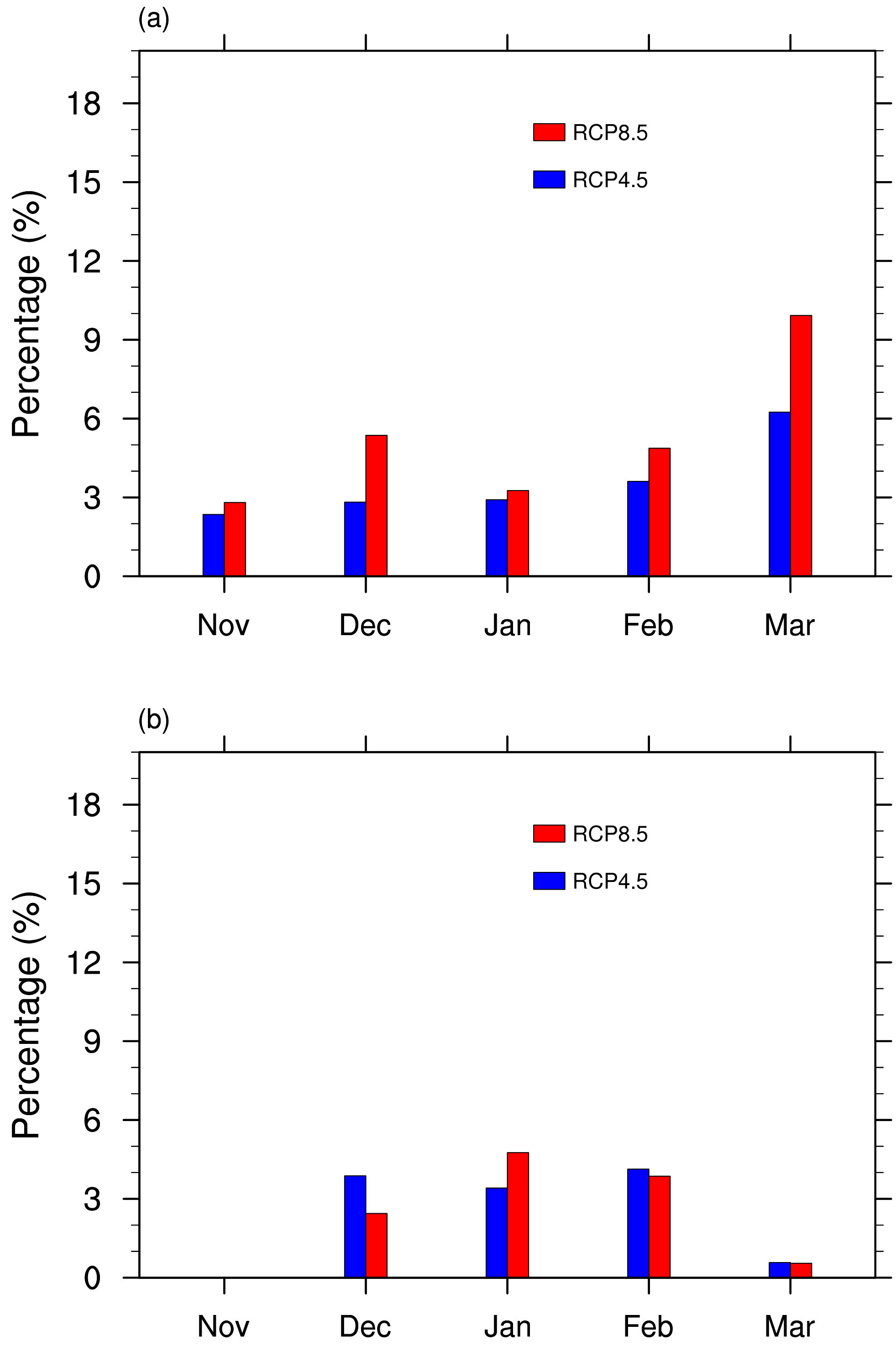

Fig. S8 The inter-GCM uncertainties on the change of area with snow frequency larger than 10% (a) and 90% (b) under the RCP4.5 (blue) and RCP8.5 (red) emission scenarios for the five months (Unit: %)

Table. S1. The ten representative stations used in this study

	Name	State	Latitude	Longitude	Elevation	Landscape type
1	Hot Springs	VA	38.00°N	79.83°W	681.5m	Mountain
2	Jefferson City	MO	38.58°N	92.18°W	204.2m	Urban
3	Danville	IL	40.14°N	87.65°W	170.1m	Inland
4	New York	NY	40.78°N	73.97°W	39.6m	Urban/Coast
5	State College	PA	40.79°N	77.87°W	356.6m	Mountain
6	Norwalk	OH	41.27°N	82.62°W	204.2m	Lakeside
7	Plymouth-Kingston	MA	41.98°N	70.70°W	13.7m	Coast
8	Keene	NH	42.94°N	72.32°W	158.5m	Inland
9	Hart	MI	43.67°N	86.42°W	234.7m	Lakeside
10	Presque Isle	ME	46.65°N	68.00°W	182.6m	Inland

Table. S2. The CMIP5 GCMs used in this study

	Model	Institution
1	CanESM2	Canadian Centre for Climate Modelling and Analysis, Canada[1]
2	CCSM4	National Center for Atmospheric Research (NCAR), USA[2]
3	CNRM-CM5	Centre National de Recherches Meteorologiques, Meteo-France, France[3]
4	CSIRO-MK3.6.0	Australian Commonwealth Scientific and Industrial Research Organization, Australia[4]
5	GFDL-CM3	NOAA Geophysical Fluid Dynamics Laboratory (GFDL), USA[5]
6	IPSL-CM5A-MR	Institut Pierre-Simon Laplace, France[6]
7	MIROC5	AORI (Atmosphere and Ocean Research Institute), NIES (National Institute for Environmental Studies), JAMSTEC (Japan Agency for Marine-Earth Science and Technology), Japan[7]
8	MPI-ESM-MR	Max Planck Institute for Meteorology, Germany[8,9]
9	MRI-CGCM3	Meteorological Research Institute, Japan[10]
10	NorESM1-M	Norwegian Climate Centre, Norway[11]

References:

1. Chylek, P., Li, J., Dubey, M. K., Wang, M., & Lesins, G. Observed and model simulated 20th century Arctic temperature variability: Canadian Earth System Model CanESM2. *Atmos. Chem. Phys. Discuss.*, **11**, 22893-22907 (2011).
2. Gent, P. R., et al. The Community Climate System Model version 4. *J. Climate*, **24**, 4973-4991 (2011).
3. Voldoire, A., et al. The CNRM-CM5.1 global climate model: description and basic evaluation. *Clim. Dyn.*, **40(9)**, 2091-2121, DOI: 10.1107/s00382-01101259-y (2012).
4. Rotsayn, L., et al. Improved simulation of Australian climate and ENSO-related climate variability in a global climate model with an interactive aerosol treatment. *Int. J. Climatology.*, **30(7)**, 1067-1088 (2010).
5. Donner, L. J., et al. The dynamical core, physical parameterizations, and basic simulation characteristics of the atmospheric component AM3 of the GFDL Global Coupled Model CM3. *J. Climate*, **24**, 3484-3519 (2011).
6. Mignot, J., & Bony, S. Presentation and analysis of the IPSL and CNRM climate models used in CMIP5. *Clim. Dyn.*, **40**, DOI: 10.1007/s00382-01301720-1 (2013).
7. Watanabe, M., et al. Improved climate simulation by MIROC5: Mean states, variability, and climate sensitivity. *J. Climate*, **23**, 6312-6335 (2010).
8. Raddatz, T. J., et al. Will the tropical land biosphere dominate the climate-carbon cycle feedback during the twenty-first century? *Clim. Dyn.*, **29**, 565-574 (2007).
9. Marsland, S. J., Haak, H., Jungclaus, J. H., Latif, M., & Röske, F. The Max-Planck-Institute global ocean/sea ice model with orthogonal curvilinear coordinates. *Ocean Modelling*, **5**, 91-127 (2003).

10. Yukimoto, S., et al. Meteorological Research Institute-Earth System Model version 1 (MRI-ESM1): Model description. Technical Reports of the Meteorological Research Institute No. 64, 2011, 83pp (2011).

11. Bentsen, M., et al. The Norwegian Earth System Model, NorESM1-M – Part 1: Description and basic evaluation. *Geoscientific Model Development Discussions*, **5**, 2843-2931 (2012).
